# En Bloc Resection of a Giant Retroperitoneal Liposarcoma: A Surgical Challenge

**DOI:** 10.7759/cureus.8730

**Published:** 2020-06-20

**Authors:** Talal Almas, Muneeb Ullah, Maryam Ehtesham, Absam Akbar, Muhammad Kashif Khan

**Affiliations:** 1 Internal Medicine, Royal College of Surgeons in Ireland, Dublin, IRL; 2 General Surgery, Maroof International Hospital, Islamabad, PAK; 3 Internal Medicine, Aga Khan University, Karachi, PAK; 4 Surgical Oncology, Federal Government Poly Clinic (Post Graduate Medical Institute), Islamabad, PAK; 5 Surgical Oncology, Maroof International Hospital, Islamabad, PAK

**Keywords:** liposarcoma, retroperitoneal, en bloc

## Abstract

Liposarcomas are exceedingly rare entities that evoke malignant transformation of connective tissue and fat cells. These tumours occur throughout the soft tissues of the body, afflicting a myriad of regions. In the adult population, liposarcomas represent the most prevalent subtype of sarcomas, and often arise de novo. Retroperitoneal liposarcomas (RLS) are a ubiquitous subset of sarcomas that, due to their deep location in the hollow abdomen, can grow to astronomical proportions before manifesting any noticeable symptoms; a prompt diagnosis of RLS is therefore often rendered dilatory. We hereby delineate the case of a 43-year-old woman who presented with vague left hemiabdominal distention and discomfort. A subsequent computed tomography scan divulged a giant retroperitoneal growth impaling on and thus displacing the pancreas. A compartmental, en bloc resection was performed, with subsequent histopathology of the excised specimen revealing a well-differentiated liposarcoma. The surgical intervention was curative and led to an uneventful recovery. This paper highlights the pertinence of surgical management as an appropriate treatment modality for a complete resection of RLS.

## Introduction

Liposarcomas are malignant tumours of mesenchymal cells that can infiltrate a plethora of soft tissues in the body. Retroperitoneal sarcomas encompass tumours that are mesenchymal in origin and arise from the retroperitoneum. Of all retroperitoneal sarcomas, retroperitoneal liposarcomas (RLS) are established to be the most prevalent [1]. Due to their location in the hollow abdomen, retroperitoneal sarcomas, including RLS, can often evade early detection, manifesting signs and symptoms characteristic of an abnormal growth only with advanced disease. Merely 10%-20% of sarcomas are believed to be retroperitoneal sarcomas [2]. Pertinently, retroperitoneal sarcomas have a high resectability rate, soaring to as high as 95% according to a study [3]. While the resection of RLS can yield fecund post-operative outcomes in a majority of the cases, an advanced tumour, one encasing adjacent organs, tissues, and structures, can present a surgical conundrum. We hereby elucidate the case of a 43-year-old female presenting with a giant RLS encasing the left kidney, splenic flexure, descending colon, and the left kidney. Due to extensive encasement of adjacent structures, an en bloc resection was deemed apt; its adept execution, however, presented an exacting surgical challenge. 

## Case presentation

**Figure 1:**
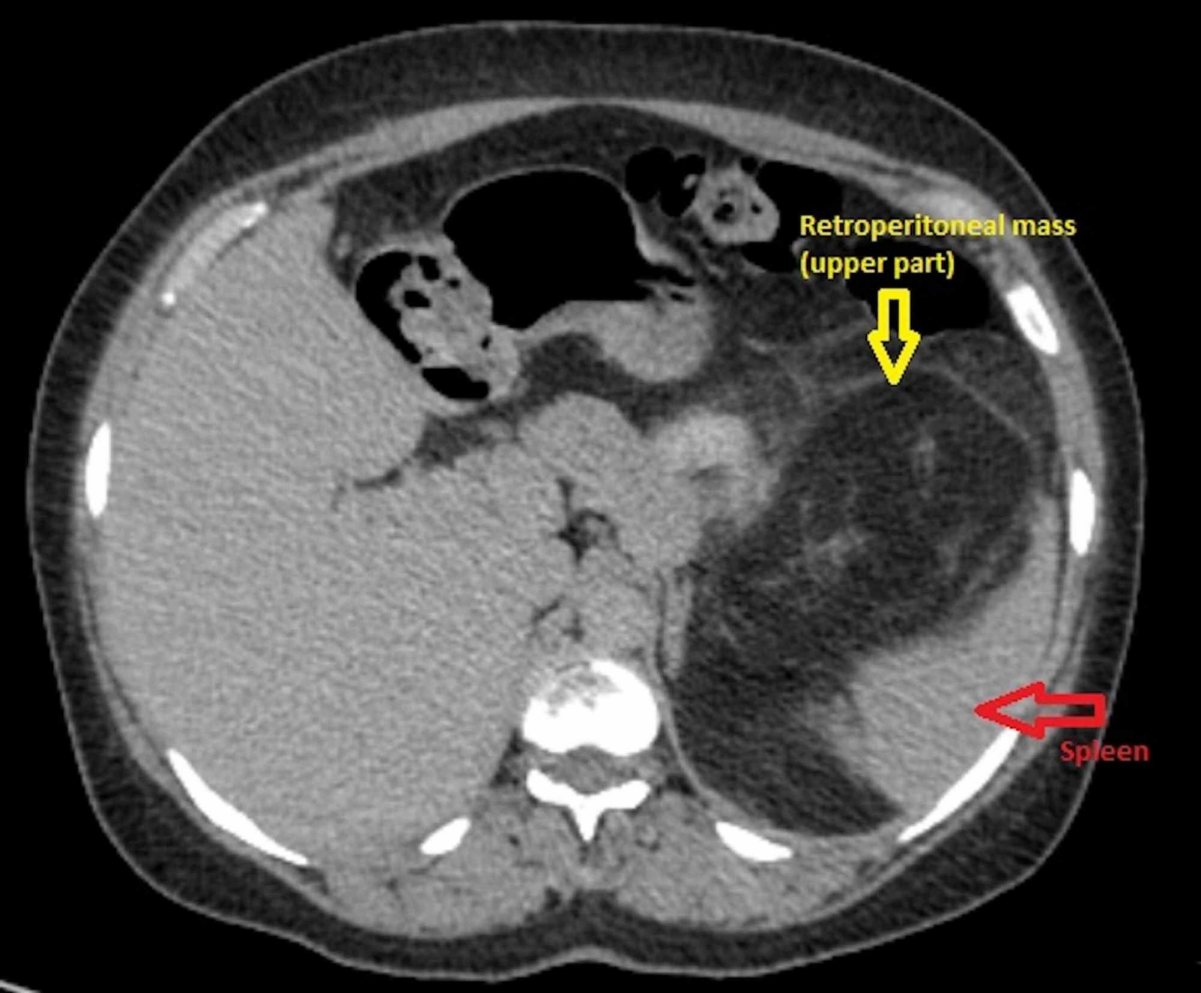
Contrast-enhanced CT scan of the abdomen and pelvis showing a retroperitoneal growth (yellow arrow) displacing the spleen (red arrow) laterally and the stomach medially.

We delineate the case of a 43-year-old female who presented to our department with a two-year history of persistent discomfort in her left abdomen and associated vague abdominal heaviness. The patient had no comorbid conditions, and her past medical and surgical histories were unremarkable. Since the inception of her symptoms, she had experienced concomitant constipation for which she had seen a physician in her remote village. Due to an acute presentation, and no associated red-flag signs, the physician attributed the constipation to the patient’s history of consuming a diet bereft of high-fibre foods. In the six months that followed, the patient began to appreciate a painful, poorly demarcated abdominal lump, for which she ultimately underwent an ultrasound one month prior to presenting to us. The ultrasound divulged an abnormal growth in the left hemiabdomen; further imaging, however, was deemed necessary in order to yield a definitive diagnosis. At presentation, the patient had a huge, palpable abdominal mass that was firm and non-tender. The mass was noted to be in the left hemiabdomen, and appeared to extend from her left hypochondrium to the left iliac fossa, ostensibly traversing the midline. Thereafter, the patient was advised to under undergo a computed tomography (CT) scan of her chest, abdomen, and pelvis. The contrast-enhanced CT scan revealed a giant retroperitoneal mass, measuring 50 x 40 x 30 cm. The CT scan showed that the giant retroperitoneal growth was boring behind the pancreas, displacing it supero-anteriorly. Furthermore, the tumour was also noted to be impaled on the splenic hilum, thereby displacing the spleen laterally (Figure 1).

**Figure 2:**
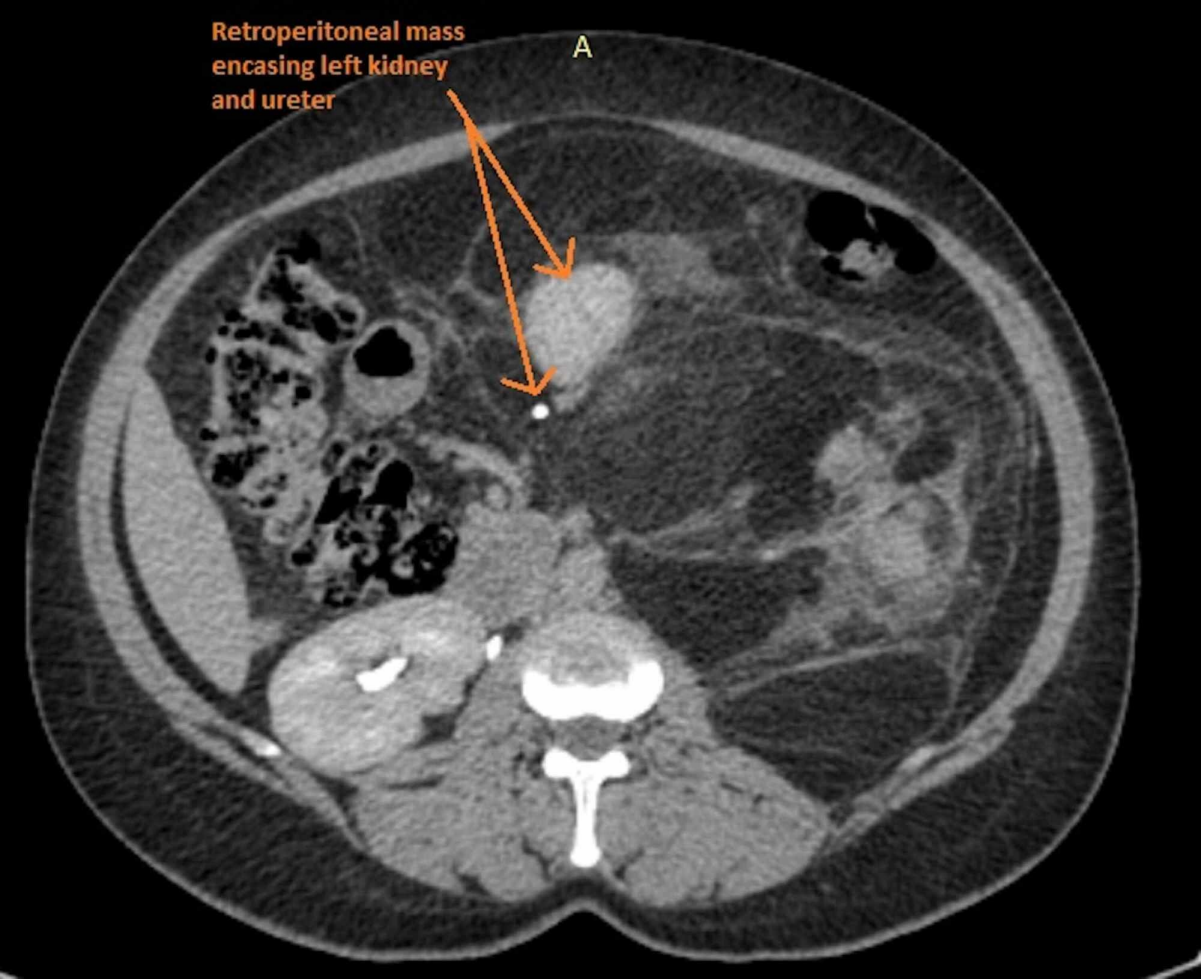
An intravenous contrast CT image showing complete encasement of her left kidney, ureter, and the descending colon by the tumour (arrows).

The CT scan further revealed that the retroperitoneal tumour was in fact completely encasing the left kidney, displacing it medially and across the midline. Moreover, based on the CT findings, it was aptly construed that the massive retroperitoneal growth was indeed crossing the midline itself, entirely encasing the splenic flexure and the descending colon (Figure 2). 

**Figure 3:**
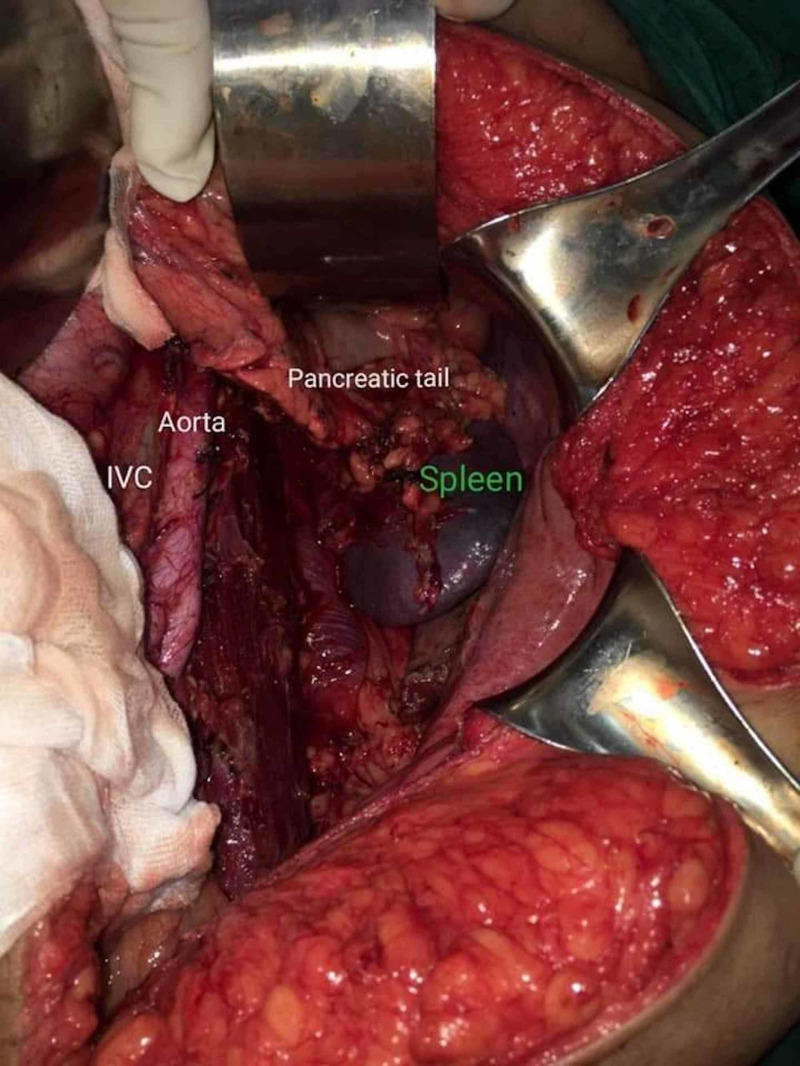
The inferior vena cava (IVC), the aorta and bifurcation into iliacs is visible; pancreatic tail and spleen, visibly clear of tumour margins, are seen after a seamless resection of the mass.

A subsequent CT-guided core biopsy revealed a well-differentiated liposarcoma. Due to the extensive encasement of adjacent structures by the tumour, the case was discussed at a multidisciplinary team meeting (MDT), which involved the pathologist, an oncological surgeon, general surgeon, and the radiologist. A surgery was promptly planned, and an en bloc compartmental resection approach for the excision of the tumour and the encased structures was performed (Figure 3). 

**Figure 4:**
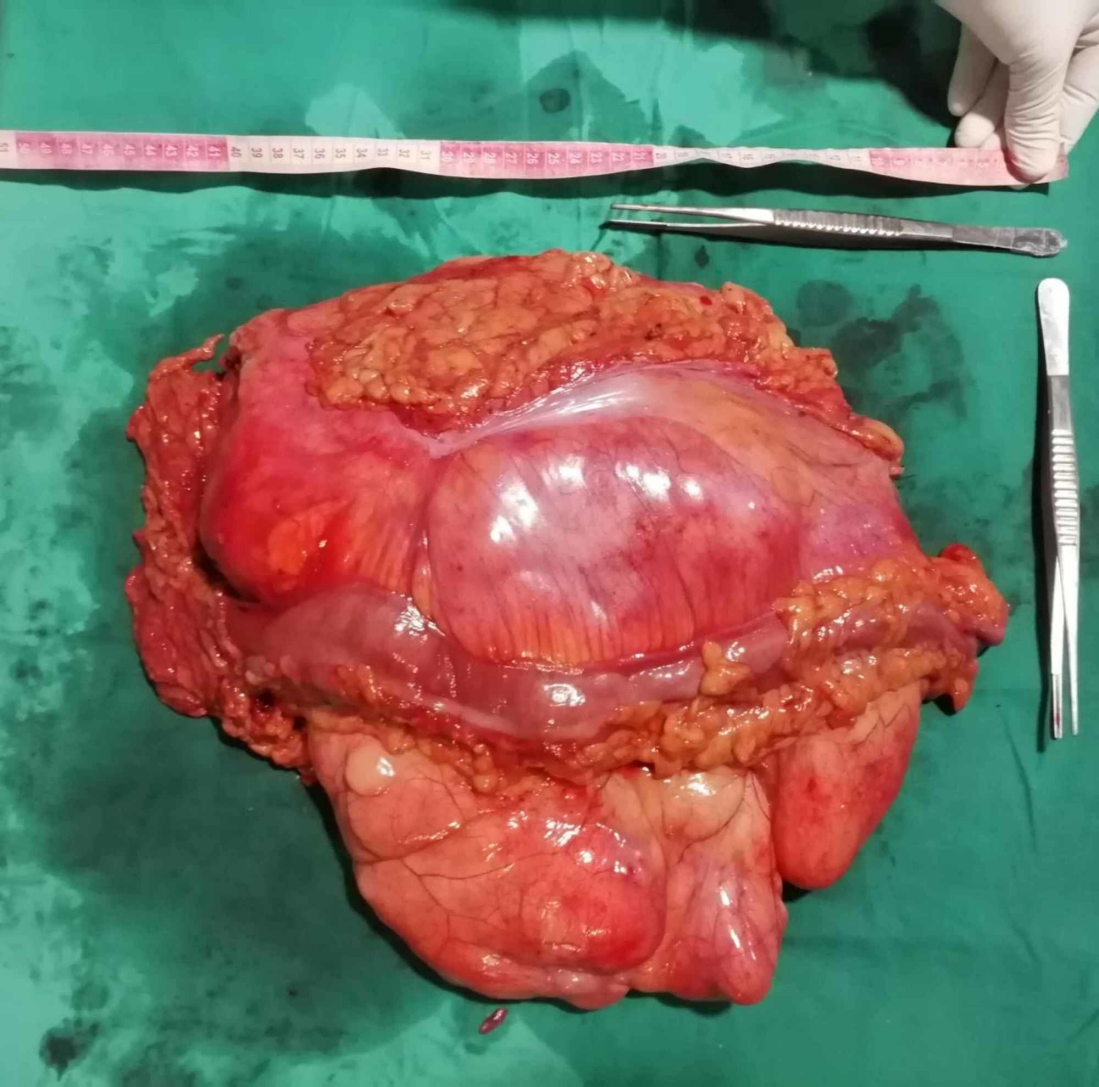
The resected en bloc specimen measuring roughly 40 x 30 cm.

A successful en bloc resection of the mass, with the encased kidney and involved colon, yielded the specimen (Figure 4). Additionally, Hartmann’s procedure was performed due to extensive colonic involvement.

The excised sample was subsequently sent for histopathology, which revealed fibroadipose and fibromuscular tissue infiltrated by an adipocytic lesion composed of variably sized mature adipocytes and bands of fibrotic stroma containing atypical spindle to stellate stromal cells. Enlarged, hyperchromatic nuclei were appreciated with prominent nucleoli and scant mitoses. The spindle cells, in particular, demonstrated mild atypia on a background of present lipoblasts. Sections examined revealed that both the attached bowel and kidney wall were tumour-free.

Post-operatively, the patient's recovery was uneventful and the patient was discharged on the fourth day post-operatively. To date, at one-year follow-up, the patient continues to thrive and do well, with no recurrence(s). 

## Discussion

Liposarcomas are specific subtypes of sarcomas that involve the soft tissues [4]. Although they can be found in a myriad of regions, liposarcomas are found in the retroperitoneal region around half the time. The tumours can be differentiated into three distinct histologic and genetic subtypes, such as dedifferentiated liposarcoma (DDLS), well-differentiated, myxoid/round cell liposarcoma, and pleomorphic liposarcoma. Regardless of the type of liposarcoma, surgical excision remains the most efficacious treatment modality [4]. These tumours present both diagnostic and therapeutic challenges as they are difficult to palpate on physical examination, and an inability to completely excise the tumour predisposes to the possibility of a recurrence. The tumour is believed to mainly afflict male patients between the ages of 40 and 60 years [5]. Although a multitude of sarcomas can be found in the retroperitoneal region, RLS remains the most common subtype [6]. The expansive anatomical spaces afforded by the retroperitoneum, along with the tendency of RLS to locally invade, mean that the tumour frequently evades detection and presents with symptoms only with advanced sizes [6]. The subtype of the tumours further governs the prognosis, with DDLS presenting with a recurrence rate of over 80% [4,6]. Well-differentiated tumours have a 100% five-year survival rate, followed by an 88% survival rate afforded by myxoid liposarcomas [7].

RLS generally present with a progressive increase in abdominal girth that does not subside [4,5]. Patients may also present with diffuse abdominal pain and fever lasting one to two weeks [6]. Upon suspicion, patients subsequently undergo CT scans of the abdomen and pelvis to determine the extent of tumour invasion. MDT consultations play a pivotal role in the management of liposarcomas [8]. Surgical excision remains the most effective treatment modality for retroperitoneal sarcomas, with diffuse involvement of the adjacent organs in the abdominal cavity necessitating en bloc resection of the tumour [8]. En bloc resection is therefore preferred and imperative in cases where large proportions of the tumours encase the abdominal cavity, infiltrating multiple organs. Previous attempts at neoadjuvant chemotherapy complementing resection have proved to be futile [6]. Post-operatively, histological analysis of the tumour is conducted, which is instrumental in confirming the diagnosis of a particular subtype of liposarcoma [9].

Despite the requirement of negative surgical margins involving resection of the pseudocapsule, en bloc resection presents a surgical quandary owing to the close proximity of the tumour to vital organs, such as the great vessels [6]. As a result, en bloc resection of RLS presents an exacting surgical challenge. Surgeons must prioritize resection based on various categories, such as considering organ involvement and involvement of the surrounding fat independently [10]. Muscle and ligament involvement also guides surgeons in assessing the degree of aggressiveness they must demonstrate while resecting [7]. Striking a balance between eradicating malignancy and observing patient safety is at the crux of surgical therapy for these tumours and determines survival outcomes for patients [11]. Therefore, an MDT analysis of each case with discussion of benefits and drawbacks of the different management options is pivotal in portending favourable post-operative outcomes [12]. Additionally, intra-operative bleeding presents a concomitant risk that comes entrenched in surgical resection and must therefore be minimized [11]. Organ failure and death post-operatively are major complications of the surgery. Furthermore, atrial fibrillation is another potentially grave complication that can ensue due to the haemodynamic shifts in blood volumes and atrial stretch during surgery [11]. Follow-up of patients for the recurrence of symptoms is therefore crucial and in cases deemed palliative, surgery targeting symptomatic relief is warranted [13]. 

## Conclusions

Due to their location in the hollow abdomen, RLS often present late in the disease course, remaining clinically silent and thus evading prompt detection. Clinical manifestations elicited by retroperitoneal sarcomas are often vague, and most often present only with exorbitant tumour growth and subsequent encasement of the adjacent structures. En bloc resection is thus warranted, presenting an exacting surgical challenge. 
